# Characterization of Within-Host *Plasmodium falciparum* Diversity Using Next-Generation Sequence Data

**DOI:** 10.1371/journal.pone.0032891

**Published:** 2012-02-29

**Authors:** Sarah Auburn, Susana Campino, Olivo Miotto, Abdoulaye A. Djimde, Issaka Zongo, Magnus Manske, Gareth Maslen, Valentina Mangano, Daniel Alcock, Bronwyn MacInnis, Kirk A. Rockett, Taane G. Clark, Ogobara K. Doumbo, Jean Bosco Ouédraogo, Dominic P. Kwiatkowski

**Affiliations:** 1 Wellcome Trust Sanger Institute, Wellcome Trust Genome Campus, Hinxton, United Kingdom; 2 Global Health Division, Menzies School of Health Research, Charles Darwin University, Darwin, Northern Territory, Australia; 3 Centre for Genomics and Global Health, University of Oxford, Oxford, United Kingdom; 4 Mahidol-Oxford Research Unit, Faculty of Tropical Medicine, Mahidol University, Bangkok, Thailand; 5 Malaria Research and Training Centre, Faculty of Medicine, University of Bamako, Bamako, Mali; 6 Institut de Recherche en Sciences de la Santé, Direction Régionale de l'Ouést, Bobo-Dioulasso, Burkina Faso; 7 Department of Public Health Sciences, Section of Parasitology, University of Rome La Sapienza, Rome, Italy; 8 Wellcome Trust Centre for Human Genetics, University of Oxford, Oxford, United Kingdom; 9 London School of Hygiene & Tropical Medicine, Keppel Street, London, United Kingdom; Université Pierre et Marie Curie, France

## Abstract

Our understanding of the composition of multi-clonal malarial infections and the epidemiological factors which shape their diversity remain poorly understood. Traditionally within-host diversity has been defined in terms of the multiplicity of infection (MOI) derived by PCR-based genotyping. Massively parallel, single molecule sequencing technologies now enable individual read counts to be derived on genome-wide datasets facilitating the development of new statistical approaches to describe within-host diversity. In this class of measures the *F_WS_* metric characterizes within-host diversity and its relationship to population level diversity. Utilizing *P. falciparum* field isolates from patients in West Africa we here explore the relationship between the traditional MOI and *F_WS_* approaches. *F_WS_* statistics were derived from read count data at 86,158 SNPs in 64 samples sequenced on the Illumina GA platform. MOI estimates were derived by PCR at the *msp-1* and *-2* loci. Significant correlations were observed between the two measures, particularly with the *msp-1* locus (*P* = 5.92×10^−5^). The *F_WS_* metric should be more robust than the PCR-based approach owing to reduced sensitivity to potential locus-specific artifacts. Furthermore the *F_WS_* metric captures information on a range of parameters which influence out-crossing risk including the number of clones (MOI), their relative proportions and genetic divergence. This approach should provide novel insights into the factors which correlate with, and shape within-host diversity.

## Introduction

Particularly in high transmission regions, individuals may carry multiple genetically distinct parasite clones in a single malaria infection (multi-clonal infection). This dynamic poses numerous challenges to malaria control. Recombination between genetically distinct parasite clones (out-crossing) is a major risk factor for generating novel parasite variants with clinically important phenotypes such as virulence, drug resistance or immune evasion. Parasite diversity dynamics also have important implications for host immune acquisition, and influence the selection and spread of drug resistance [1], [2], [3], [4], [5]. Furthermore, the intense competition enabled between distinct clones within an infection appears to promote the evolution of highly virulent *Plasmodium* parasites [6], [7], [8], [9]. Numerous studies have sought correlations between parasite multi-clonality and clinical phenotypes such as patient age, immune status, clinical outcome, drug sensitivity, gametocyte production and gametocyte infectivity [10], [11], [12], [13], [14], [15], [16], [17]. However, these studies have been constrained by the limited availability of tools to effectively gauge the genetic composition of multi-clonal infections. Constrained in part by these limitations we still have only a poor understanding of how within-host diversity is shaped in different epidemiological settings.

Our current understanding is that multi-clonal infections may be generated by super-infection (the composition of multiple infectious bites with genetically distinct clones), and that this may be a frequent occurrence in high transmission areas. However, it has also been demonstrated that a single mosquito inoculation may carry a large amount of parasite diversity [18], and that this again may be dependent on the population level diversity. In addition, within-host dynamics may be shaped by factors such as host immunity and density-dependent control mechanisms [19]. In order to better understand the dynamics of multiple-clone infections and parasite out-crossing, effective tools to address within-host diversity within the context of the population-level diversity are essential.

Traditionally, within-host parasite diversity has been described by the number of distinct clones within an infection, or, multiplicity of infection (MOI). A common approach is the use of PCR to derive the number of repeat length variants observed at a few highly diverse loci, most frequently variable number tandem repeats (VNTRs) in the genes encoding antigens such as the merozoite surface protein 1 (MSP1) and 2 (MSP2) [20], [21]. The large repertoire of alleles at a single VNTR locus is practical for finger-printing malaria parasites, particularly for drug surveillance. However, limitations on the number and “nature” of the loci constrain effective characterization of within-host diversity. Furthermore, as the clone counting approach does not account for the population level of diversity, limited insight is gained into the risks of out-crossing in a given population.

Massively parallel sequencing technologies now enable characterization of parasite diversity at high depth and molecular resolution [22], [23], [24]. Individually, SNPs do not capture as much diversity as VNTRs, and so the clone counting MOI method is not effective for SNP data. Alternatively, the single molecule sequencing approach of platforms such as the Illumina Genome Analyzer enables individual allele counts to be derived at each SNP position, and the opportunity for new statistical approaches to describe within-host parasite diversity based on population genetics such as the *F_WS_* metric (Manske, Miotto et al., in preparation). This metric characterizes not just within-host diversity, but also its relationship to local population diversity, essentially measuring the risk of out-crossing/inbreeding.

Here, using PCR at the *msp1* and *2* loci, we explore the relationship between the traditional VNTR-based MOI and genome-wide, SNP-based *F_WS_* metric in clinical (non-cultured) *Plasmodium falciparum* samples from malaria-endemic regions in West Africa.

## Results and Discussion

### PCR-based estimation of MOI

The traditional method for characterizing within-host diversity in *P. falciparum* entails MOI estimation using genotype data from the *msp-1* and -*2* loci [20]. This approach utilizes family specific and repeat length variants in each gene to differentiate parasite clones (see Methods). In the West African samples, genotyping success rates at the *msp-1* and *-2* loci were 100% (64/64) and 98% (63/64), respectively. The distributions of MOI estimates for each assay/population are presented in Figure S1. A summary of these distributions is presented in Table 1. MOI estimates were slightly higher in Burkina Faso (mean = 2.95) than Mali (mean = 2.57), but the difference was not significant (t = 1.28, *P* = 0.209). These estimates are similar to previous observations in Mali and Burkina Faso [25], [26]. In the combined population (West Africa), the mean MOI estimate was 2.83. The gene-specific MOI estimates demonstrated similar distributions to the maximum MOI (Figures S2, 3), although *msp-2* estimates (mean = 2.54) were moderately higher than *msp-1* (mean = 2.19) (t = −1.82; *P* = 0.071). Moderate gene-specific differences were also observed between populations. The *msp-1* MOI estimates were higher in Burkina Faso (mean = 2.33) than Mali (mean = 1.90), with borderline significance (t = 1.94, *P* = 0.059). The *msp-2* MOI estimates were only moderately higher in Burkina Faso (mean = 2.64) than Mali (mean = 2.33), and the difference was not significant (t = 0.91, *P* = 0.368). In summary, the MSP MOI data indicated high multi-clonality in West Africa, with slightly higher levels in Burkina Faso than Mali, and subtle differences in the estimates derived from the *msp-1* and -*2* loci.

**Table 1:** Summary of MOI and *F_WS_* estimates in Burkina Faso, Mali and the combined (West Africa) population.

	Burkina Faso	Mali	All (West Africa)
Mean Maximum MOI (range)	2.95 (1–5)	2.57 (1–4)	2.82 (1–5)
Mean MSP1 MOI (range)	2.33 (1–4)	1.91 (1–4)	2.19 (1–4)
Mean MSP2 MOI (range)	2.64 (1–5)	2.33 (1–4)	2.54 (1–5)
Mean *F_WS_* (range)	0.69 (0.19–1.00)	0.81 (0.32–1.00)	0.73 (0.19–1.00)

### Genome-wide characterization of within-host diversity (*F_WS_*)

The *F_WS_* metric describes the relationship between the diversity observed within a patient to that of the population using estimations of heterozygosity (i.e. the probability that two randomly selected parasites carry different alleles at a given locus). In any given population, heterozygosity is dependent on the allele frequencies at that locus, as described by the Hardy-Weinberg principle, and is therefore influenced by the level of out-crossing in the population. By combining allele counts across all samples in the population, we can derive an estimate of population allele frequencies at each locus, and consequently estimate heterozygosity at the population level (*H_S_*). Similarly, for each sample, using allele counts at each locus, we can estimate within-host heterozygosity (*H_W_*), enabling computation of the *F_WS_* measure. Essentially, the *F_WS_* metric provides a measure of the risk of out-crossing between the parasites within an individual to generate new genotypes during recombination in the mosquito host. Thus, the metric captures information on all of the sample parameters which influence the risk of new parasite genotypes being generated at meiosis. These include not just the overall diversity within an individual but also the level of similarity between the parasites and their relative proportions. A low *F_WS_* reflects a low risk of inbreeding/high risk of out-crossing and thus high within-host diversity. Whilst simple calculations of the heterozygosity at each locus within a sample provide some level of information on within-host diversity, the ability to describe this diversity in the context of the level of diversity in the population is critical to capturing information on the risk of out-crossing. Consequently, population level heterozygosity estimates are essential for effective computation and interpretation of the *F_WS_* metric.

We previously described *F_WS_* distributions in a range of *P. falciparum* populations, demonstrating global patterns of within-host diversity consistent with our knowledge of the epidemiology and human (and parasite) migration patterns in the regions studied (Manske, Miotto *et al.*, in preparation). In the West African samples addressed here, a mean *F_WS_* of 0.73 was observed, with 28% samples exhibiting “high” *F_WS_* estimates (i.e. ≥0.95: see Figure S2). In each of Mali and Burkina Faso, the mean *F_WS_* scores were 0.81 (29% ≥0.95) and 0.69 (30% ≥0.95) respectively. The low proportion of samples with high *F_WS_* scores is indicative of a large degree of panmixis (low sub-structure) amongst the parasites in the populations sampled here. Presumably, super-infection with genetically distinct parasites is a relatively frequent occurrence. It follows that the risks of parasite out-crossing, and consequent threat to malaria control efforts, are moderately high in these populations.

In accordance with the *msp*-based results, greater within-host diversity (lower *F_WS_*) was observed in Burkina Faso (mean = 0.69) than Mali (mean = 0.81) (t = −2.08, *P* = 0.042). This observation may reflect subtle differences in the epidemiology of the sites studied here, such as rural (Mali) versus urban (Burkina Faso) influences on transmission dynamics [27], [28]. Further exploration of the *F_WS_* profile in different epidemiological settings is required to address this and other putative determinants of within-host parasite diversity. This should also enable more effective interpretation of the relative out-crossing risks associated with different *F_WS_* scores.

A wide range of *F_WS_* scores were observed in the West African populations (Figure S2). Thus, even after gauging within-host diversity estimates in terms of the out-crossing risk and related parameters (number, relative proportion, and degree of divergence between clones), there appears to be heterogeneity in within-host diversity in the West African populations. It remains to be determined how well this heterogeneity reflects the heterogeneity in clinical outcomes such as disease severity, gametocyte prevalence and infectivity, patient age and immune status, in West Africa and other populations. To date, the parasite genetic basis of these outcomes has generally only been addressed with regard to clone count measures of within-host diversity (review in [29]). The extent to which the clone counting MOI approach reflected the additional diversity parameters captured by the *F_WS_* metric remained uncertain. We therefore explored this relationship further.

### Relationship between genome-wide *F_WS_* metric and MSP1+MSP2 MOI estimates

**Figure 1:**
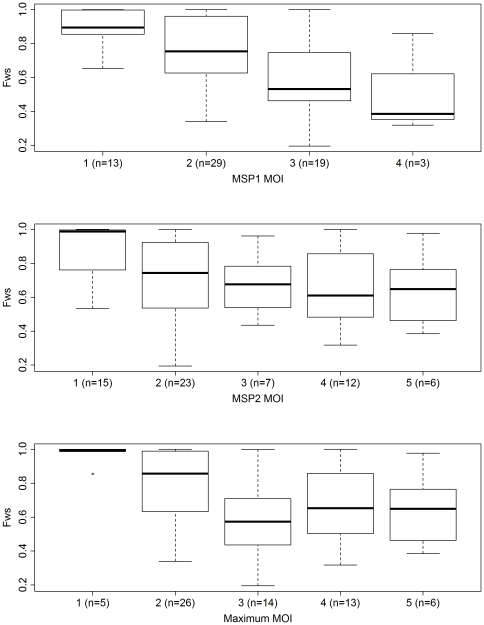
*F_WS_* against MSP1 MOI, MSP2 MOI, and maximum MOI.

Using the *F_WS_* score as a proxy to genome-wide within-host SNP diversity, we assessed the correlation between this metric and the MSP-based MOI estimates. A significant negative correlation was observed between the MSP1 MOI and *F_WS_* (rho = −0.480, *P* = 5.92×10^−5^), as illustrated in Figure 1. The *F_WS_* correlations with each of the MSP2 and maximum MOI estimates were less strong but both remained significant (rho = −0.366, *P* = 0.003; rho = −0.384, *P* = 0.002, respectively) (Figure 1). These correlations demonstrated that the MOI and *F_WS_* metric broadly agreed on their interpretations of within-host diversity. However, differences in two key areas, 1) the analytical approach, and 2) the number and nature of loci examined, may underlie the remaining deviations between the two measures.

A moderate proportion of samples (13/64 [20.3%]) demonstrated multiple clones by MSP genotyping but appeared to be largely “clonal” with respect to the *F_WS_* (≥0.95). These results presumably reflect infections with multiple clones exhibiting limited divergence and thus limited within-host diversity. As detailed below, any of a number of differences, both technical and analytical, between the PCR-based MOI and *F_WS_* approaches may underlie these discrepancies. Fewer samples (4/64 [6.25%]) exhibited a clonal MSP outcome concurrent with high diversity *F_WS_* (≤0.70). The ability to capture the relatedness between the clones within an infection should facilitate our understanding of the impact of within-host regulatory effects such as inter-parasite competition and host immunity on parasite diversity dynamics.

In addition to the core analytical approach, the PCR-based MOI approach may be constrained in ability to capture the complexity of an individual infection owing to sensitivity to the number of loci examined, the level of polymorphism at each locus, family-specific versus intra-family variation and selective pressures at one or more loci. In these respects, the genome-wide, SNP-based, *F_WS_* approach is more robust. In general, even with highly diverse loci, more markers ensure a more accurate representation of diversity [30]. Indeed, a larger number of multiple clone infections were detected by the maximum MOI (92% [59/64]) than with the *msp-1* (79% [50/63]) and *-2* (77% [49/64]) loci individually. However, practicalities limit the number of loci that can be examined by PCR.

Owing to differences in the level and nature of polymorphism, inter-locus variation in diversity is not unexpected within a multi-clonal malaria infection. Indeed, the *msp-1* and *-2* genes exhibited modest differences in polymorphism, reflected in their different strengths of correlation with the *F_WS_* metric (Figure 1). Inter-locus differences may also result from locus-specific selective pressures which are not representative of the genome as a whole. This is a potential limitation of MSP1 and 2, which appear to be under selective pressure from the host immune system [31].

The distinction of alleles at VNTR loci by electrophoretic mobility is another potential limitation of the PCR-based approach, as differences in sequence composition between equally migrating fragments may be missed. Furthermore, it is generally accepted that VNTRs evolve at different rates and under different mechanisms from SNPs [32], [33], [34], [35]. Thus, VNTRs may not provide an effective representation of the SNP diversity in the genome. Rather, as observed with the moderately high frequency (20.3%) of isolates demonstrating largely clonal dynamics with *F_WS_* estimates but polyclonal dynamics at the MSP loci, VNTR-based estimates may overestimate the true genome-wide within-host diversity.

A potential shortfall of the *F_WS_* approach is the integrity of the genome-wide dataset. A highly stringent SNP discovery and allele-calling process was employed here to ensure high confidence in the dataset (see Methods). In addition, only good quality samples with high sequence depth were included (Figure S3). The comparable sensitivity of the PCR-based and genome-sequencing approach has yet to be determined. However, in contrast to the potential ambiguity in detecting and distinguishing bands on gels, the ability to count individual reads in Illumina sequence data enables more objective allele-calling.

Juliano and colleagues achieved extensive sequence depth at the *msp-1* and *-2* loci by focusing 454 sequencing on amplicons of these two genes alone [36]. Using the clone-counting approach, facilitated by haplotype information, an average of ∼5 more *msp-1* and *-2* variants were detected relative to the genotyping data. As with the PCR-based MOI method, this approach is powerful for distinguishing clones in clinical drug trials. For characterization of within-host diversity however, the use of just 2 VNTR loci under potential selective pressure renders this approach sensitive to the features discussed above. With continual improvements in read length and sequence yield on high-throughput, single-molecule sequencing platforms, haplotype-based clone counting approaches similar to those described by Juliano and colleagues should be feasible on a broader, genome-wide, scale.

### Conclusion

The traditional approach to estimating MOI using VNTRs at a handful of loci such as *msp-1* and *-2* is a simple and moderately cheap method with demonstrated practicality for finger-printing clones in drug trials. However, genome-wide measures such as the *F_WS_* statistic, by capturing information not just on the number of clones in a sample but also on their respective ratios and the degree of inter-clone variation at hundreds of thousands of polymorphic positions, provide a greater wealth of information on within-host diversity which is highly robust against potential locus-specific artifacts. With rapidly advancing progress in whole genome sequencing technologies, including ever increasing read lengths and continual reductions in cost, this approach is now feasible in hundreds of samples. The ability to assess within-host diversity in genome-wide datasets from non-cultured (clinical) samples sourced from low resource field settings, as demonstrated here, should revolutionize clinical and epidemiological studies of *Plasmodium*.

## Methods

### Samples

Samples were collected from field sites in Mali and Burkina Faso within the framework of a large, multi-center *P. falciparum* genome sequencing project to facilitate SNP discovery and population genetic characterization (Manske, Miotto *et al.*, in preparation). Consenting patients of all ages and ethnic groups presenting at the clinic with symptoms of uncomplicated malaria and *P. falciparum* parasitaemia were recruited to the study. In Mali, samples were collected from clinics in two rural villages (Kolle and Faladje). In Burkina Faso, samples were collected from permanent health dispensaries in three suburbs of Bobo-Dioulasso (Colsamma, Ouezzinville, and Sakaby). Details of the sample processing procedures in the laboratory are described elsewhere [37]. Briefly, venous blood (2–8 ml) was processed to deplete the human white blood cell fraction, and DNA extraction was undertaken using Qiagen QIAamp blood extraction kits as per the manufacturer's instructions.

### Ethics

Informed, written consent was obtained from patients over 18 years of age and from a parent or guardian for younger patients. The study was approved by the Comite d'Ethique de la Faculté de Médecine de Pharmacie et d'Odontostomatologie, Bamako, Mali, and Comite d'Ethique Institutionnel du Centre Muraz, Bobo- Dioulasso, Burkina Faso.

### Sequencing and SNP Calling

Sixty-four samples (21 Mali, 43 Burkina Faso) with >500 ng total DNA and <60% human DNA contamination were sequenced on the Illumina Genome Analyzer platform. Details of the sequencing, SNP discovery and genotype calling process are described elsewhere (Manske, Miotto *et al.*, in preparation). Briefly, up to 6 lanes were sequenced per sample, with 37, 54 or 76 bp paired-end reads. Coverage distributions are presented in Figure S3. The “raw” sequence data for the samples can be accessed in the European Nucleotide Archive (www.ebi.ac.uk/ena/data/search/?query=plasmodium). For each sample, sequence data from multiple lanes was merged, and mapped to the *P. falciparum* reference genome (3D7 version 2.1.5; ftp://ftp.sanger.ac.uk/pub/pathogens/Plasmodium/falciaprum/3D7/3D7.version2.1.5) using the *bwa* program (available from http://bio-bwa.sourceforge.net) with the default parameters [38]. The resulting alignments were processed in *samtools* (http://samtools.sourceforge.net/cns0.shtml) using the default parameters to identify positions with one or more bases differing from the reference sequence (*putative* SNPs). Genotypes were called at 86,158 high confidence SNP positions (*typable* SNPs) identified by a stringent quality-filtering SNP discovery process detailed elsewhere (Manske, Miotto *et al.*, in preparation). This SNP list is referred to as version 1gamma and is available on the MapSeq database (www.mapseq.net/pf). For the purpose of assigning confident genotype calls to the dataset, all genotypes in the version 1 gamma dataset with less than 5 reads were assigned a status of missing data.

### Calculation of *F_WS_*

Details of the underlying basis for calculations used to derive the *F_ws_* metric are described elsewhere (Manske, Miotto *et al.*, in preparation). The *F_WS_* metric was calculated for each individual sample, using the formula *F_WS_* = 1−(*Hw*/*Hs*), where *Hw* is the within-individual heterozygosity and *Hs* is the within-population heterozygosity. At each biallelic SNP, heterozygosity was estimated using the formula *H* = 1−(*p* ^2^+*q* ^2^), where *p* and *q* are the frequencies of the two alleles (*p* = 1−*q*). At each SNP, *p* and *q* were estimated for each individual as the proportions of sequencing reads that carried each allele in the individual sample. At population level, the allele frequencies at the SNP were estimated as the mean of the allele frequencies in the individuals comprising the population sample; the frequency of the least common allele at that SNP in the West African population was noted as the *minor allele frequency* (MAF). Since heterozygosity depends on allele frequencies, each of the 86,158 SNPs was assigned to one of ten equally-sized MAF intervals ([0.0–0.05], [0.05–0.1] … [0.45–0.5]). Estimates of *Hw* and *Hs* were calculated for each MAF interval, as the means of the *Hw* and *Hs* across all SNPs in that interval. For each individual, *Hw* estimates were plotted against the corresponding *Hs* estimates for all MAF intervals, and the slope of the resulting straight-line plot was used to evaluate the *Hw*/*Hs* ratio, and thus compute *F_WS_* for the individual.

### Genotype-based MOI Estimation

The traditional method of genotyping in the *P. falciparum* merozoite surface protein - 1 (*msp-1*) and -2 (*msp-2*) genes was undertaken on 64 of the typable samples using a nested PCR approach [20]. This method utilises family specific variation and variation in repeat length in each gene to distinguish parasite clones. In general, each parasite will exhibit one of three family-specific sequences, K1, R033 or MAD20, in the *msp-1* gene, and one of two family-specific sequences, FC27 or IC/3D7, in the *msp-2* gene. Within each family-specific sequence, regions comprising repeat-length polymorphisms enable further distinction of clones. The number of distinct bands observed in each sample was summed across the families in each of the *msp-1* and *-2* genes separately, providing gene-specific MOI estimates. The maximum MOI estimate was then derived as the maximum gene-specific MOI estimate for each sample. The standard Welch two-sample t-test was used to measure the significance of difference in MOI estimates between populations and assays using R software.

## Supporting Information

Figure S1 **MSP-based MOI Estimates.**
(TIFF)

Figure S2 **Distribution of** ***F_ws_*** **scores in the West African samples**. Dashed lines indicate thresholds for highly diverse (*F_ws_*≤0.7) and moderately “clonal” (*F_ws_*≥0.95) samples.
(TIFF)

Figure S3 **Read depth distribution at the typable SNP positions in the West African samples.** Distribution of SNP coverage at read depth (number of sequenced nucleotides covering a given locus) thresholds of 1, 5, 10, 15, 20 and 25.
(TIFF)
