# Supplementary figures and images for: Characterization of Within-Host Plasmodium falciparum Diversity Using Next-Generation Sequence Data
Source: PLoS One. 2012 Feb 29;7(2):e32891. doi: 10.1371/journal.pone.0032891 (PMC3290604; doi:10.1371/journal.pone.0032891)

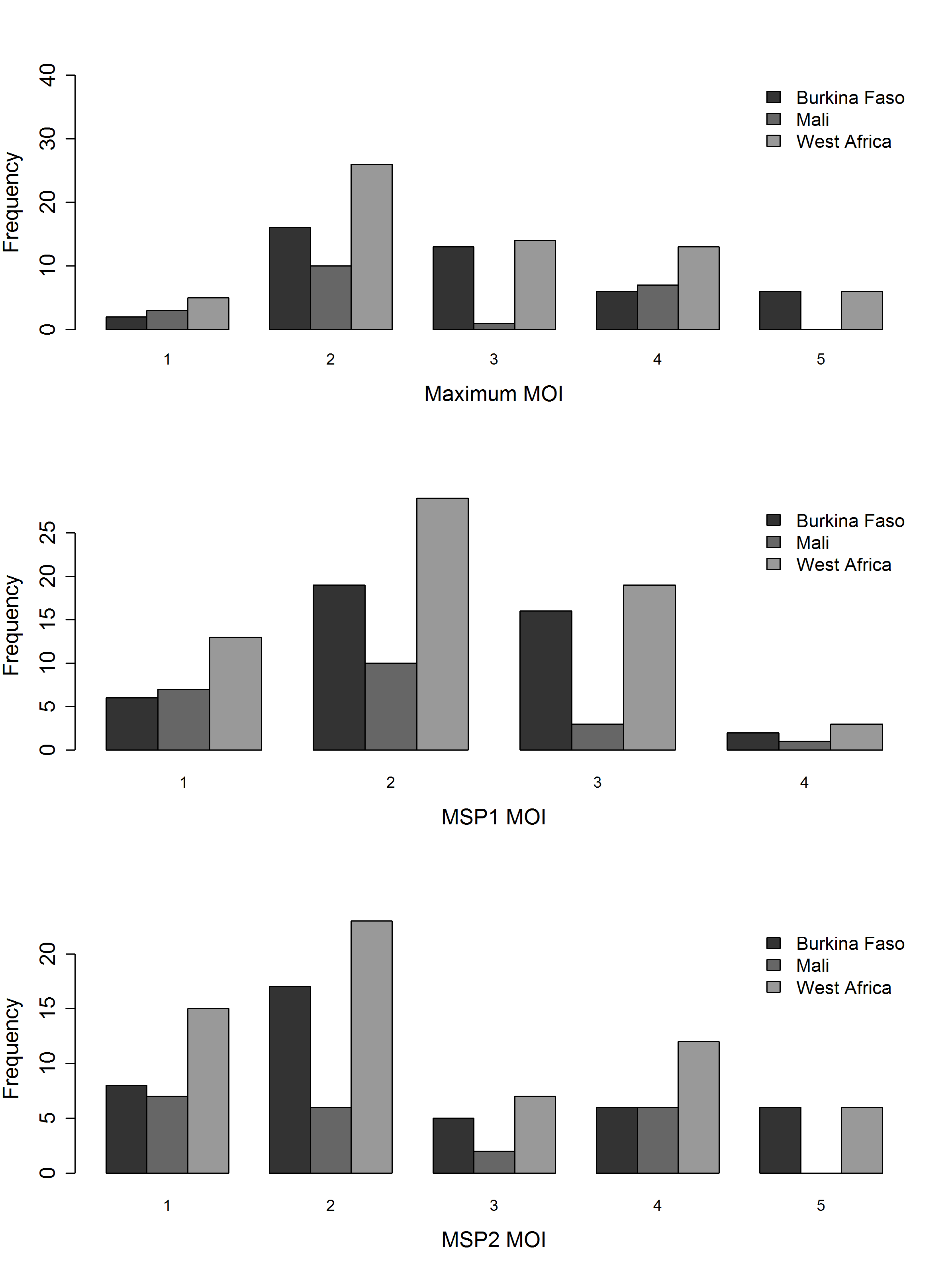

Supplement: Figure S1 — MSP-based MOI Estimates. (TIFF) [file pone.0032891.s001.tiff]

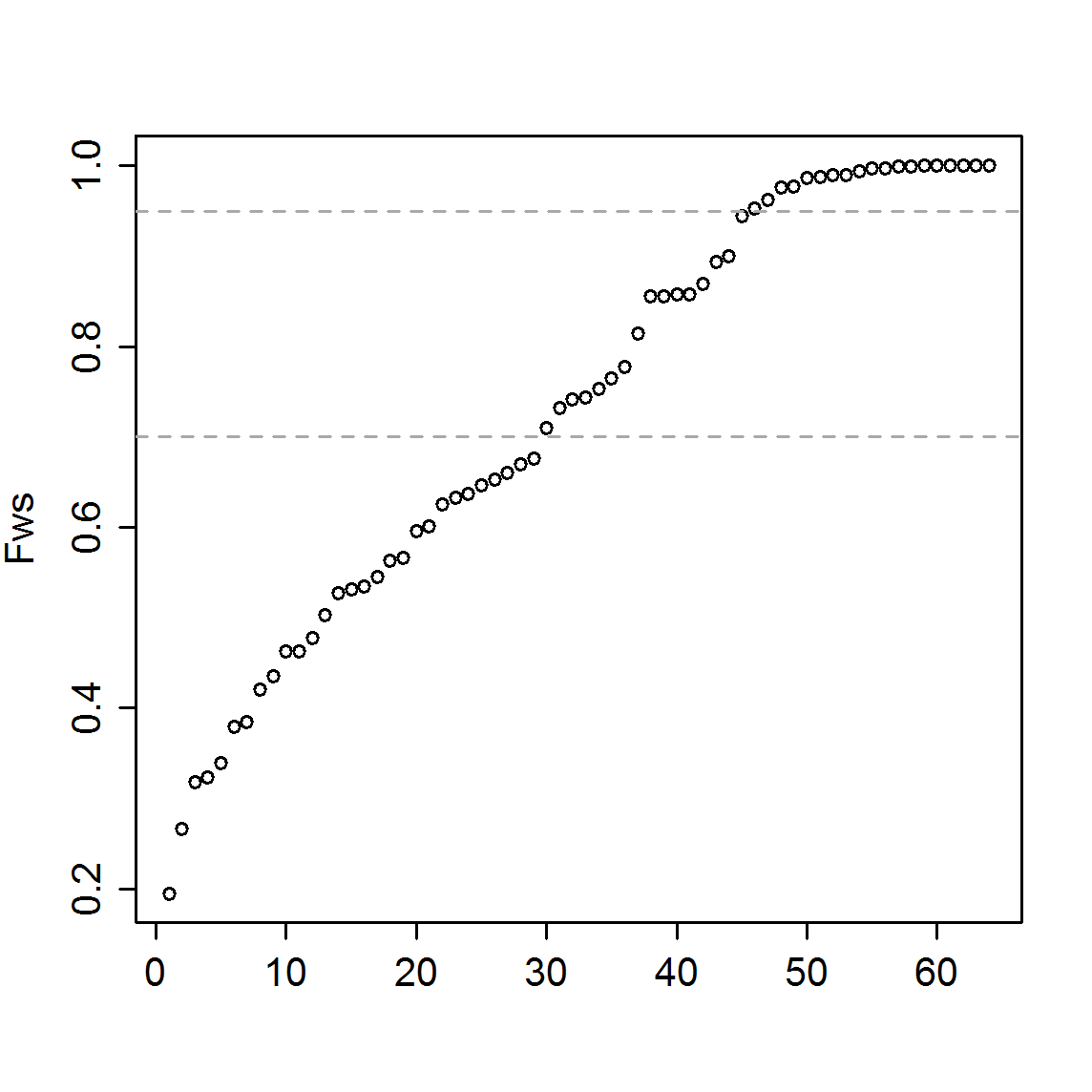

Supplement: Figure S2 — Distribution of Fws scores in the West African samples. Dashed lines indicate thresholds for highly diverse (Fws≤0.7) and moderately “clonal” (Fws≥0.95) samples. (TIFF) [file pone.0032891.s002.tiff]

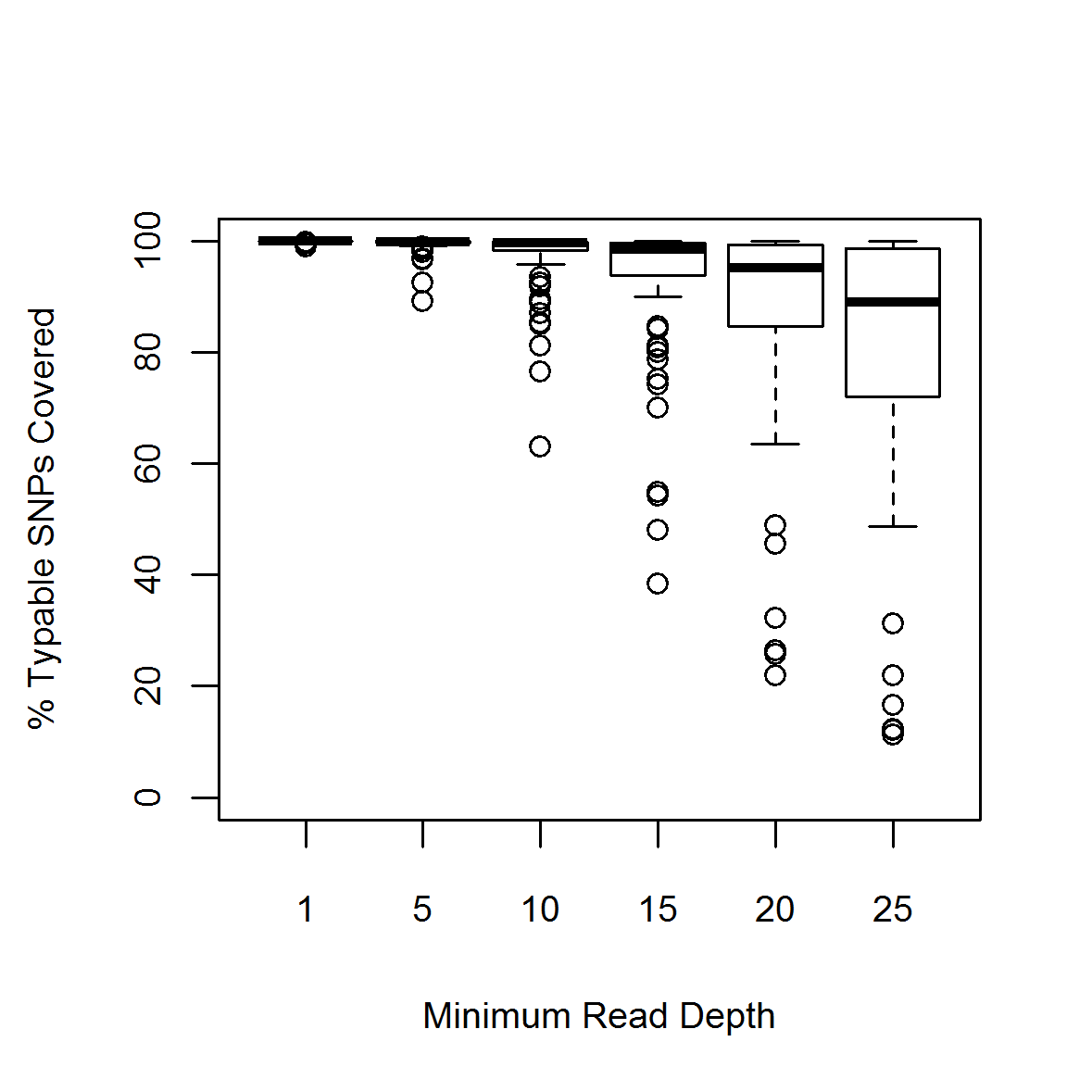

Supplement: Figure S3 — Read depth distribution at the typable SNP positions in the West African samples. Distribution of SNP coverage at read depth (number of sequenced nucleotides covering a given locus) thresholds of 1, 5, 10, 15, 20 and 25. (TIFF) [file pone.0032891.s003.tiff]
